# Supplementary material for: Mechanism and structural diversity of exoribonuclease-resistant RNA structures in flaviviral RNAs
Source: Nat Commun. 2018 Jan 9;9:119. doi: 10.1038/s41467-017-02604-y (PMC5760640; doi:10.1038/s41467-017-02604-y)
Supplement: Supplementary file 1 — Supplementary Information [file 41467_2017_2604_MOESM1_ESM.pdf]

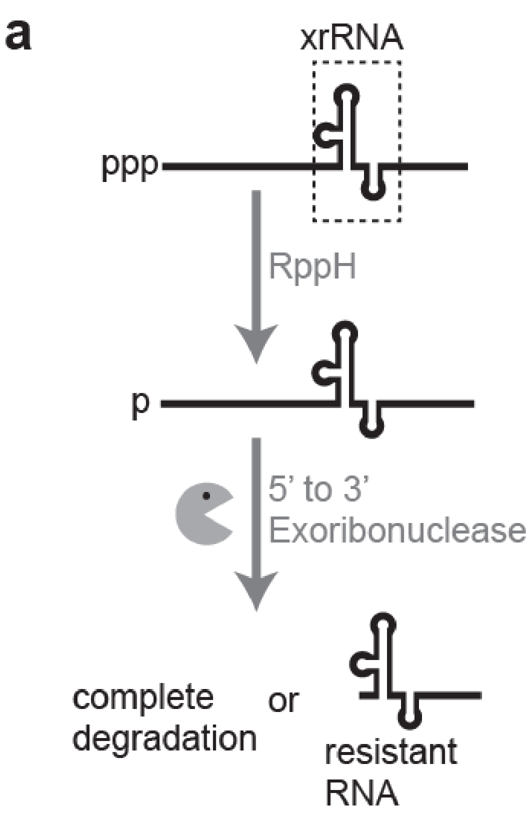

Supplementary Figure 1. Schematic of the previously described *in vitro* assay. (**a**) xrRNA is treated with RNA 5' Pyrophosphohydrolase (RppH) to convert the 5’ triphosphate to monophosphate, allowing exoribonuclease to load and degrade. Exoribonuclease resistance due to RNA structures results in a degradation intermediate rather than complete degradation. **(b)** SDS-PAGE analysis of purified exoribonucleases.

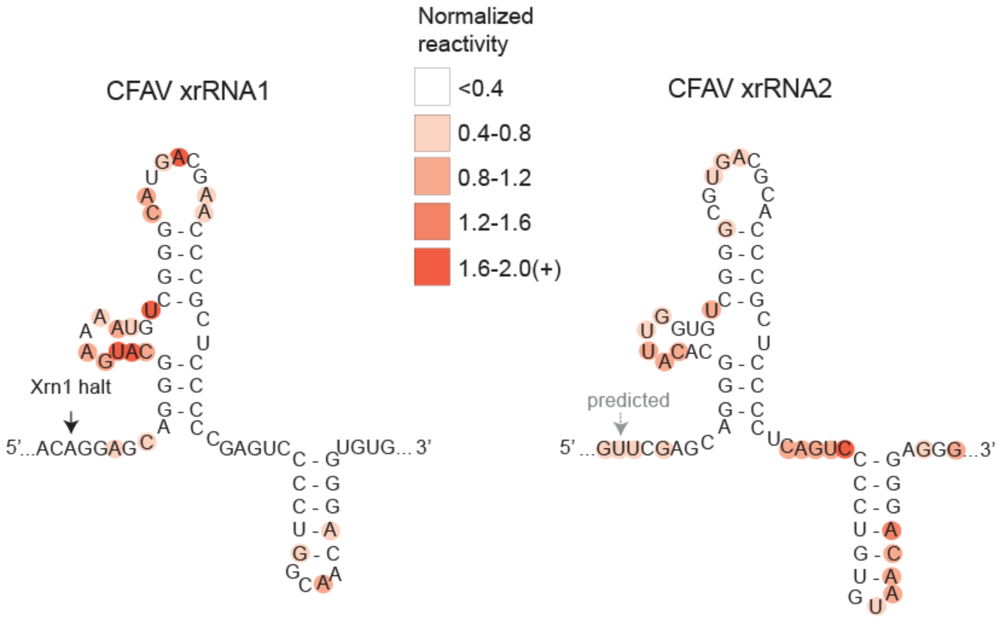

Supplementary Figure 2. Secondary structure models of CFAV xrRNAs. The mapped or predicted Xrn1 halt site is indicated. Nucleotides are shaded to indicate their normalized SHAPE reactivity.

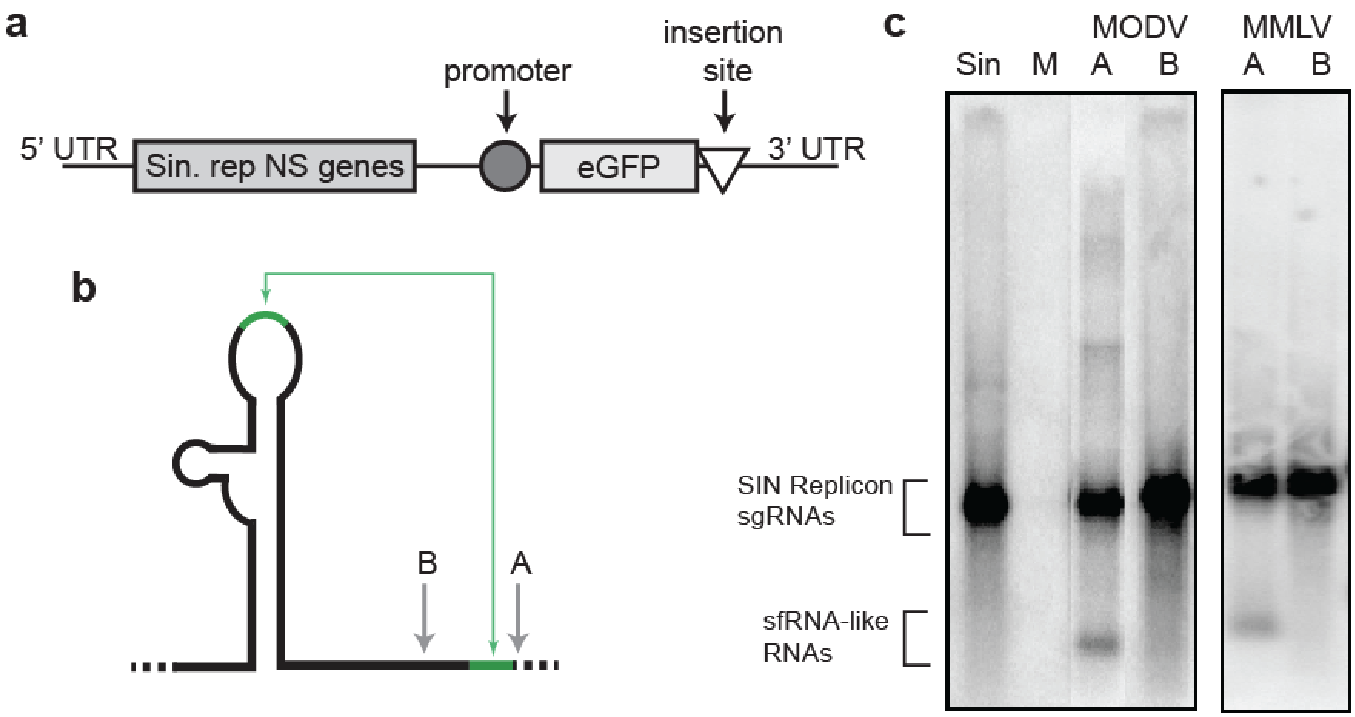

Supplementary Figure 3. Long-range pseudoknot interaction for NKVFV xrRNA function. **(a)** Design of the pSinrep5eGFP replicon vector; a complete description of this vector is contained in the Methods section. **(b)** Cartoon representation of the secondary structure of an xrRNA from the NKVFV. The green sections denote the putative long-range base-pairing interaction. The 3’UTR of MMLV and MODV was cloned into the replicon vector, with two versions of the xrRNA. One contained the downstream half of the long-range interaction (“A”), the other had this sequence deleted (“B”) to prevent formation of the long-range interaction within the context of the full 3’UTR. **(c)** Northern blot analysis of total RNA isolated from BHK-21J cells transfected with the replicon vector containing the A and B versions of the 3’UTRs of MODV and MMLV. The subgenomic RNA produced from the replicon and the sfRNA-like RNAs are labeled. Deletion of the downstream half of the putative long-range interaction causes a loss of sfRNA-like RNAs. SIN = replicon without the KNVFV 3’UTRs, M = Mock infection.

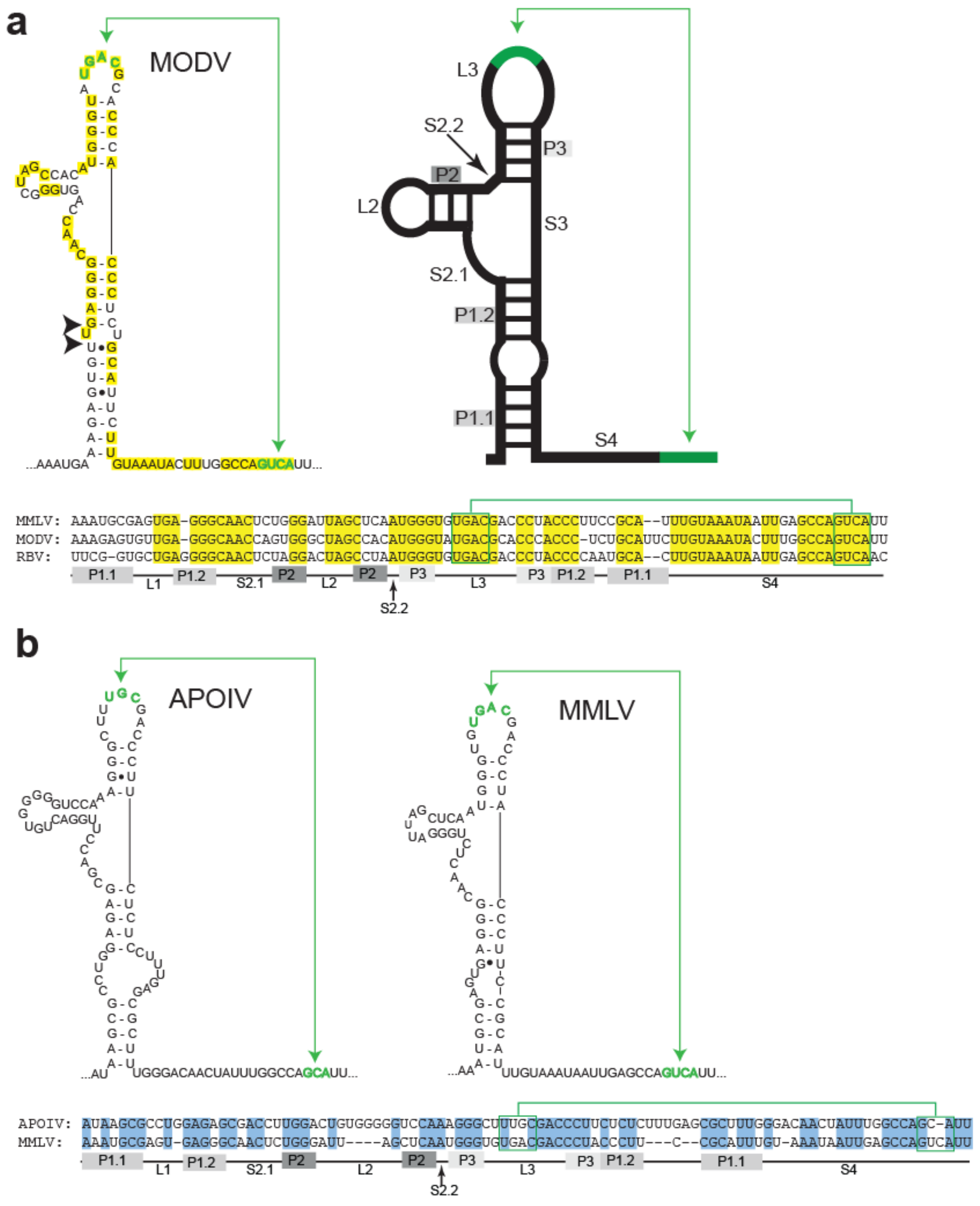

Supplementary Figure 4. Sequence alignments of NKVFV xrRNAs. (**a**) Sequence alignments of NKVFV xrRNAs. Alignment of xrRNAs from MODV (NC_003635), MMLV (NC_004119), and Rio Bravo virus (RBV; AF452050). Yellow shading indicates absolutely conserved nucleotides. The location of the long-range base-pairing is shown with gren lines and boxes. The locations of secondary structural elements are indicated below the alignments. At left, the secondary structure of the MODV xrRNA, with absolutely conserved nucleotides and secondary structure elements labeled. (**b**) Sequence alignment of MMLV with a putative xrRNA from the 3'UTR of NKVFV Apoi virus (APOIV). Nucleotides conserved between these two viruses are shaded cyan, long-range base-pairing pseudoknot indicated in green. The sequence of the xrRNA from APOIV is more divergent but clearly can adopt a structure type that matches MMLV, MODV and RBV. In both panels (a) and (b), the precise boundaries of the secondary structure elements may vary between viruses. TBEV Western (NC_001672) was the sequence used in the chemical probing experiments (Fig. 5a) and is the reference for identifying these secondary structure elements.

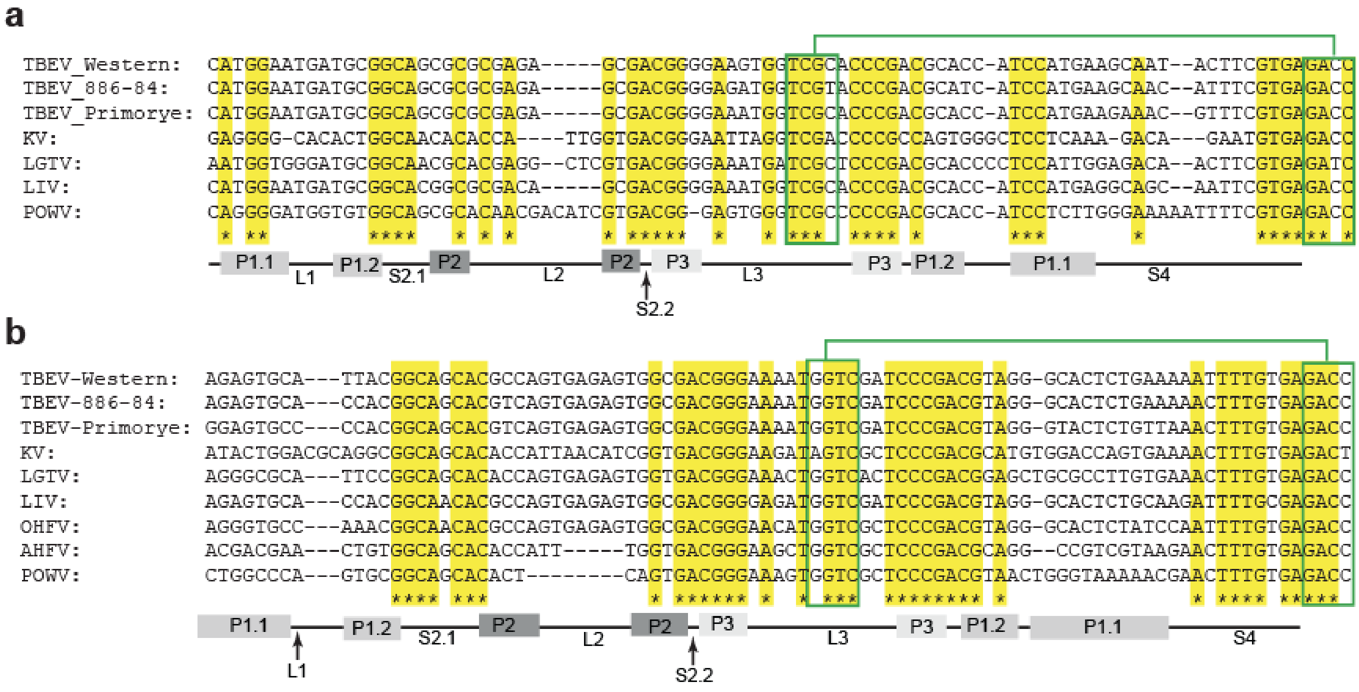

Supplementary Figure 5. Sequence alignments of TBFV xrRNAs. (**a**) Sequence alignment of the xrRNA1 from a variety of TBFVs. LGTV: Langat virus (NC_003690), TBEV: tick borne encephalitis (NC_001672, EF469662, EU816453), LIV: Louping ill virus (NC_001809), POWV: Powassan virus (NC_003687), KV:, Karshi virus (NC_006947), OHFV: Omsk hemorrhagic fever virus (NC_005062), AHFV: Alkhurma hemorrhagic fever virus (NC_004355). A similar alignment was previously published (Schnettler et al. 2014). Yellow shading indicates absolutely conserved nucleotides. The location of the long-range base-pairing is shown with green lines and boxes. The locations of secondary structural elements are indicated below the alignments. (**b**) Identical to panel (a), with xrRNA2 from a variety of TBFVs. The locations of secondary structural elements are indicated below the alignments. In both panels (a) and (b), the precise boundaries of the secondary structure elements may vary between viruses. Again TBEV_Western (NC_001672) was the sequence used in the chemical probing experiments (**Fig. 5a**) and is the reference for identifying these secondary structure elements.

Supplementary Table 1. Oligonucleotides that were used to identify and characterize the sfRNAs of NKV flaviviruses and CFAV.

The oligonucleotides that were used in this study as well as the virus towards they were directed, the NCBI file that was used to obtain the sequence and the actual nucleotide sequence of the oligonucleotides are indicated. All the oligonucleotides are complementary to the viral genome. Abbreviations in the column “Used for” refer to Northern blotting and hybridizations (Hyb.), primer extension (Prim.Ex.)

**Oligonucleotide**	**Virus**	**NCBI Accesion Number**	**Sequence**	**Used for**
NKV002	MMLV	AJ299445	CCGCTCAATCTCGAGAGGAGCGA	Hyb/Prim.Ex.
NKV003	APOIV	AF452050	CTCAGGCGCTAAAGGATGCCGCTA	Hyb.
NKV004	MODV	AJ242984	GGGTCTCCACTAACCTCTAGTCCT	Hyb.
NKV006	APOI	AF452050	CGCTCAAAGAGAGAAGGGTCGC	Hyb.
NKV019	RBV	AF452049	ACTCGGTCAGTTGGGATCATCCCAC	Hyb.
NKV020	MODV	AJ242984	CCCTAACCTATTTACAATGACTGGC	Hyb./Prim.Ex.
NKV021	CFAV	NC_008604	AGATGGGCCGCCACCACCATCTTAG	Hyb./Prim.Ex.
NKV031	RBV	AF452049	CACCCTATCAGGGTTGACTGGCTCA	Prim.Ex.
NKV033	APOIV	AF452050	CCCACTGGAATGCAATGCTGGCC	Prim.Ex.

Supplementary Table 2. Oligonucleotides that were used in the construction of the Sinrep5 constructs containing the stalling sites of MODV and MMLV.

The viral sequence (MODV or MMLV) is indicated in red

**Oligonucleotide**	**Virus**	**NCBI Accesion Number**	**Sequence**	**Polarity**	**Sinrep construct**
NKV025	MODV	AJ242984	GCGACGCGTTTAAATGAAAGAGTGTTGAGGGC	+	MODV A and B
NKV027	APOI	AJ242984	GCGCATGCGGCCAAAGTATTTACAAGAATGC	-	MODV B
NKV058	MODV	AJ242984	GCGCATGCACAATGACTGGCCAAAGTATTTAC	-	MODV A
NKV028	MMLV	AJ299445	GCGACGCGTCAACTAATGGAAAAAATGCG	+	MMLV A and B
NKV030	MMLV	AJ299445	GCGCATGCTGGCCTCAATTATTTACAAATGCGG	-	MMLV B
NKV061	MMLV	AJ299445	GCGCATGCGGAAATGACTGGCTCAATTATTTAC	-	MMLV A
